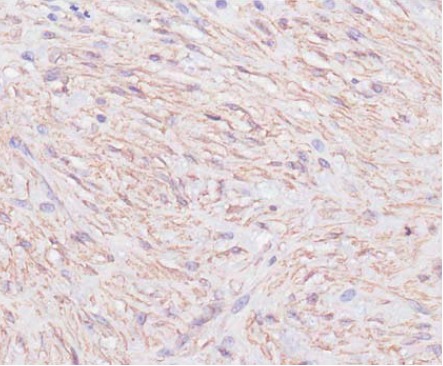# Intra-articular Nodular Fasciitis: An Unexpected Diagnosis for a Joint Lesion: A Case Report

**DOI:** 10.5704/MOJ.1407.013

**Published:** 2014-07

**Authors:** MF Michelle Chan, KO Ong, SS Leon Foo, S Selvarajan

**Affiliations:** Department of Pathology, Singapore General Hospital, Singapore; Department of Pathology, Singapore General Hospital, Singapore; Department of Pathology, Singapore General Hospital, Singapore; Department of Pathology, Singapore General Hospital, Singapore

## Abstract

Pathological lesions in and around a joint can arise from underlying dermis, subcutis, deep muscle, bone or synovium. Clinical presentation can include joint pain, joint swelling, palpable masses and mechanical restriction. Whilst giant cell tumour of tendon sheath, pigmented villonodular synovitis, synovial chondromatosis, lipoma arborescens, juxta articular myxomas and inflammatory arthritis are the better-known conditions of the joint. Intra-articular nodular fasciitis, on the other hand, is less well recognized both clinically and radiologically. It is rarely seen in routine practice and is only described in case reports in the literature. Due to the non-specific clinical and radiological findings as well as the unfamiliarity with the entity, the diagnosis of intra-articular nodular fasciitis is usually clinched only after histological examination. We present a case of intra-articular nodular fasciitis arising in the knee joint which was not suspected clinically or radiologically.

**Key Words:**

Intra-articular, nodular fasciitis, joint, knee, MRI

## Introduction

Nodular fasciitis is a well-known benign reactive proliferation which typically presents with a history of a rapidly growing single subcutaneous mass, usually in the upper limbs or trunk with men and women being equally affected. It can also occur in dermis, muscle or intravascular locations. It tends to occur in adults who are between 20 to 50 years of age. There is sometimes an association with previous trauma. Local excision is usually curative with a low rate of recurrence. When present in infants and children, nodular fasciitis usually affects the head and neck region. Interestingly, nodular fasciitis has also been reported in a list of other unusual anatomical locations such as the parotid gland and urinary bladder. Of these rare sites, the joint is one addition. Here we present a case of an intra-articular nodular fasciitis which followed a history of trauma.

## Case Report

A 17 year-old Chinese male was referred to our outpatient clinic by a general practitioner, for a persistent right knee effusion after a fall during Chinese wushu martial arts two months earlier. He had no other significant past medical history. There was associated knee pain with limited extension and flexion. There was no locking or any instability of the joint. No palpable mass or lymphadenopathy was identified and the joint was not warm to touch.

**Fig. 1B: Corresponding T2-weighted axial MRI image with fat suppression:**
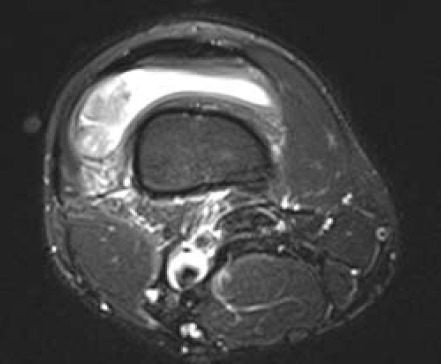


There was a 3.2x3.1x1.5cm lobulated lesion in the lateral aspect of the suprapatellar pouch (Figure 1A,1B). It displayed intermediate signal on T1-weighted and T2- weighted (fat suppression) images with avid enhancement following intravenous contrast. Hypointense foci on gradient sequence suggestive of hemosiderin were also seen in the lesion. There was a moderate right knee effusion.

An arthrotomy of the right knee was performed, showing severe synovitis with a large yellowish serous effusion and a superolateral mass in the suprapatellar pouch. The lesion was covered by and just deep to the synovium. It was localised and did not appear to involve the deep structures such as the periosteum. The lesion was excised and a partial synovectomy of the suprapatellar pouch was performed.

Macroscopically, the mass was polypoid in appearance and had a smooth external surface and a tan-white homogeneous soft cut surface. On microscopic examination, the lesion was unencapsulated and well circumscribed (Figure 2A). It was centred directly underneath the synovium and was variably cellular. It was composed of short fascicles of spindle cells arranged in a “tissue culture-like” fashion with accompanying extravasation of red blood cells (Figure 2B). There was no significant nuclear atypia. The mitotic count was up to 8 per 10 high power fields. MIB-1 proliferation index was around 10 to 20%. Focal areas of hyalinization and myxoid change were noted. The spindle cells were diffusely immunoreactive for SMA (Figure 3) and focally positive for H-caldesmon, whilst being negative for desmin and S100. The overall features were those of a nodular fasciitis.

The patient has been on six monthly follow-up for 17 months so far and is free of recurrence. This has been ascertained with follow-up MRI. He has returned to his martial art activities although unable to participate at competitive levels yet. He has a good range of knee movements and is fully functional.

## Discussion

The initial impression on clinical examination of the right knee in this patient was that of haemarthrosis secondary to possible ligament tear or an osteochondral fracture. However, based on the persistent knee effusion and MRI suggestion of haemosiderin deposits, the working diagnosis was changed to pigmented villonodular synovitis. Intranodular nodular fasciitis was not considered and the correct diagnosis was reached only after histological examination.

Whilst intra-articular pigmented villonodular synovitis, juxta-articular myxomas, synovial chondromatosis, lipoma arborescens and fibroma of tendon sheath are well recognised entities, intra-articular nodular fasciitis is rarely encountered and therefore not usually considered clinically during the investigation of joint symptoms. To date, there are 18 cases of intra-articular nodular fasciitis reported. Of these, 13 were in the knee, two in the hand, one in the ankle and two in the shoulder. All were reported in the English literature except two in the Japanese literature. Those patients in the English literature, presented with joint swelling, extension restriction, palpable masses, joint effusion and haemarthrosis. Their ages ranged from nine months to 52 years with a median age of 29 years. The average duration of their symptoms prior to presentation ranged from two months to one year. There were no recurrences during follow up. Nodular fasciitis was not considered in the differential diagnosis during the clinical or radiological work-up in any of these cases. Ten of the cases were part of a case series by Hornick and Fletcher in 2006 ^1^. They highlighted the knee as the commonest joint to be affected by intra-articular nodular fasciitis. They also noted that intra-articular nodular fasciitis tended to have a longer preoperative history than the usual subcutaneous or intramuscular variants. Similar to our case, three of the 16 cases in the English literature reported a history of trauma before the development of intra-articular nodular fasciitis^1-4^. In the case report by Yamamoto. et al ^5^, an initial misdiagnosis of malignant fibrous histiocytoma was made with the patient receiving unnecessary irradiation. This illustrates the need for greater awareness of intraarticular nodular fasciitis even for pathologists so that the patients can be managed appropriately. Whilst intraarticular nodular fasciitis has a low recurrence rate after wide excision, diffuse pigmented villonodular synovitis has a high recurrence rate even after extensive synovectomy.

In conclusion, the key learning point is not to assume every intra-articular mass in the knee with a persistent effusion and heterogeneous appearance on MRI with hemosiderin deposits to be pigmented villonodular synovitis. It is important to keep in mind rarer pathologies such as intraarticular nodular fasciitis, especially with a preceding history of trauma.

**Fig. 1A: Contrast enhanced T1-weighted axial MRI image with fat suppression showing the intra-articular lobulated mass in the suprapatellar pouch with accompanying effusion.**
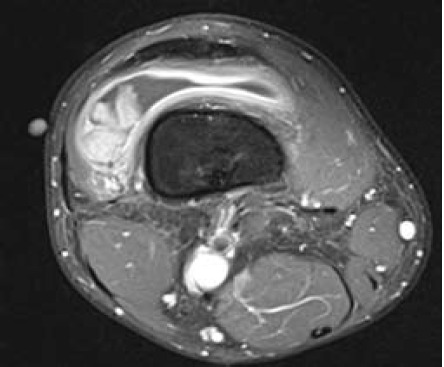


**Fig. 2A: Haemotoxylin & eosin, x 100. This low power view shows the lobulated and uncapsulated nature of the lesion.**
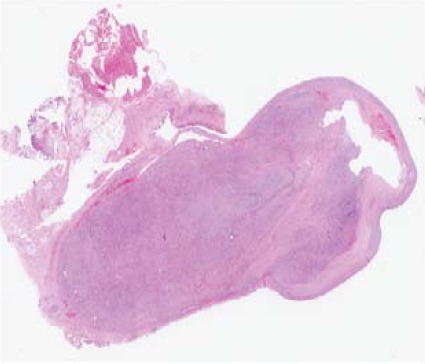


**Fig. 2B: Haemotoxylin & eosin, x 200. Typical tissue culture-like arrangement of bland spindle cells of nodular fasciitis.**
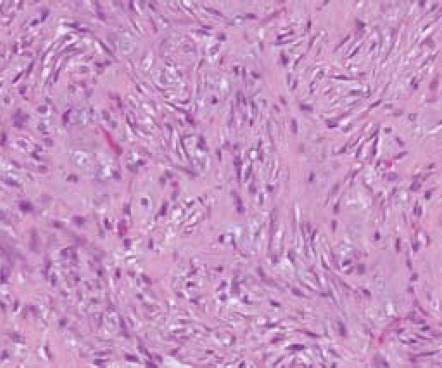


**Fig. 3: Smooth muscle actin (SMA) x 200. Spindle cells showing SMA immunoreactivity.**